# Tricuspid Regurgitation After Leadless Pacemaker Implantation: The Role of Atrioventricular Synchrony

**DOI:** 10.1002/clc.70413

**Published:** 2026-07-16

**Authors:** Zachary Alvis Seal, Debanshu Roy, Jorge Alejandro Irizarry Caro, Ivan Alfredo Mijares Rojas, Osamah Basil Ibrahim Altaee, Binish Qureshi, Tam Nguyen, Robert Chilton

**Affiliations:** ^1^ UT Health San Antonio San Antonio Texas USA

**Keywords:** atrioventricular synchrony, cardiac pacing, conduction system pacing, leadless pacemaker, Micra AV, Micra VR, tricuspid regurgitation

## Abstract

**Background:**

Tricuspid regurgitation (TR) is a recognized complication of cardiac implantable electronic devices (CIEDs), driven by both mechanical lead interference and pacing‐induced dyssynchrony. Leadless pacemakers eliminate transvalvular leads; however, the role of atrioventricular (AV) synchrony in mitigating TR progression remains unclear.

**Methods:**

This narrative review was informed by a structured literature search of PubMed, EMBASE, and the Cochrane Library through June 2025 using the search terms “leadless pacemaker,” “Micra,” “tricuspid regurgitation,” “atrioventricular synchrony,” and “conduction system pacing.” Original studies, systematic reviews, and major guideline or review articles relevant to leadless pacing and TR were considered. Articles were selected based on relevance to the review objectives rather than predefined systematic review criteria. Formal risk‐of‐bias assessment or quality appraisal was not performed.

**Findings:**

Leadless pacemakers generally demonstrate neutral effects on TR severity compared with baseline, although worsening of TR has been reported in approximately 9%–33% of patients across observational studies. Available evidence suggests that loss of AV synchrony is associated with TR progression independent of device type; however, pacing‐associated TR is multifactorial and likely also reflects ventricular dyssynchrony, right ventricular pacing burden, papillary muscle discoordination, right ventricular dysfunction, and progressive right‐heart remodeling. Direct comparative data evaluating Micra AV versus Micra VR with respect to TR outcomes remain unavailable.

**Conclusions:**

AV synchrony is biologically plausible for mitigating TR progression, but direct comparative evidence between Micra AV and Micra VR is lacking. Emerging dual‐chamber leadless systems may improve preservation of AV synchrony while maintaining the advantages of leadless pacing, although their long‐term effects on TR remain to be established. Prospective studies incorporating standardized echocardiographic endpoints are needed.

## Graphical abstract

Leadless pacemakers eliminate transvalvular lead interference but do not eliminate functional mechanisms of tricuspid regurgitation. Atrioventricular synchrony is one important, potentially modifiable contributor within a multifactorial process that also includes ventricular dyssynchrony, right ventricular remodeling, and pacing burden.

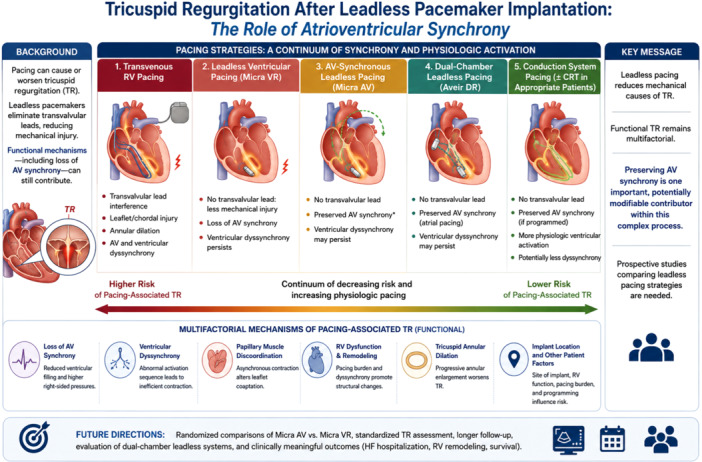

## 1 Introduction

Tricuspid regurgitation (TR) is increasingly recognized as a clinically significant complication following cardiac implantable electronic device (CIED) implantation. Transvenous right ventricular (RV) leads may contribute to TR through leaflet impingement, chordal disruption, and progressive annular dilation secondary to pacing‐induced ventricular dyssynchrony [1, 2]. Approximately 25%–29% of patients with permanent pacemakers develop TR, with device‐related mechanisms implicated in 7%–45% of cases [2]. Importantly, worsening TR following transvenous pacemaker implantation is associated with increased heart failure hospitalization and mortality (HR 1.60, 95% CI 1.28–2.00) [3].

Leadless pacemakers, such as the Micra Transcatheter Pacing System, were developed to eliminate transvalvular leads and reduce device‐related complications. However, emerging evidence suggests that TR progression may still occur despite the absence of a transvalvular lead, implicating additional mechanisms including RV pacing burden, ventricular dyssynchrony, and loss of atrioventricular (AV) synchrony [4, 5]. Pacing‐associated TR should be viewed as a multifactorial process in which mechanical lead effects, AV dyssynchrony, ventricular activation abnormalities, RV pacing burden, papillary muscle discoordination, and progressive right‐heart remodeling interact to varying degrees depending on patient characteristics and pacing modality.

The development of AV‐synchronous leadless pacing with Micra AV represents a potential advance over ventricular‐only pacing with Micra VR. By restoring AV synchrony via accelerometer‐based atrial mechanical sensing, Micra AV may improve ventricular filling and reduce right‐sided pressures [6]. However, no direct head‐to‐head comparison between Micra AV and Micra VR for TR outcomes currently exists.

**Figure 1:**
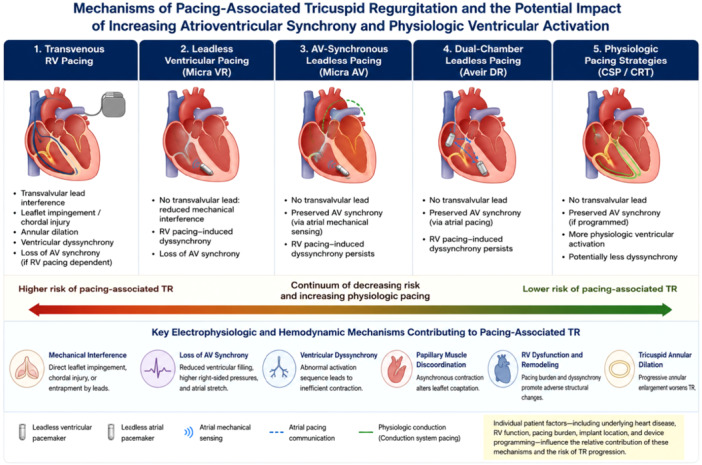
Mechanisms of pacing‐associated tricuspid regurgitation and the potential impact of increased atrioventricular synchrony and physiologic ventricular activation. Transvenous pacing leads may contribute to tricuspid regurgitation through direct interference with tricuspid valve leaflet motion, including impingement. Leadless pacing avoids transvalvular lead interference; however, ventricular pacing may still contribute to TR through interacting electrophysiologic and hemodynamic mechanisms including AV dyssynchrony, ventricular dyssynchrony, RV pacing burden, papillary muscle discoordination, RV dysfunction, and progressive right‐heart remodeling. Pacing systems are shown along a conceptual continuum of increasing preservation of atrioventricular synchrony and, where applicable, physiologic ventricular activation, from single‐chamber ventricular leadless pacing to atrioventricular‐synchronous leadless pacing, dual‐chamber leadless pacing, and traditional dual‐chamber transvenous pacing. AV, atrioventricular; RA, right atrial; RV, right ventricular; TR, tricuspid regurgitation.

This narrative review synthesizes current evidence regarding TR progression after leadless pacemaker implantation and critically evaluates the potential role of AV synchrony, while highlighting key gaps in the literature. Importantly, as pacing technologies evolve toward increasingly physiologic strategies, understanding the relative contributions of mechanical versus functional mechanisms of TR has direct implications for device selection and long‐term patient outcomes (Figure 1).

## 2 Methods

This narrative review was informed by a structured literature search of PubMed, EMBASE, and the Cochrane Library through June 2025 using the search terms “leadless pacemaker,” “Micra,” “tricuspid regurgitation,” “atrioventricular synchrony,” and “conduction system pacing.” Original investigations, observational studies, systematic reviews, meta‐analyses, and major guideline or state‐of‐the‐art review articles relevant to leadless pacing and TR were considered. Additional publications were identified through manual review of reference lists from key articles.

Studies were selected based on their relevance to the objectives of this narrative review rather than predefined systematic review eligibility criteria. Particular emphasis was placed on studies reporting echocardiographic outcomes following leadless pacemaker implantation, AV synchrony, ventricular synchrony, conduction system pacing (CSP), and mechanisms of pacing‐associated TR. No language restrictions were applied. Because this was a narrative review, a formal risk‐of‐bias assessment or quality appraisal of included studies was not performed. Preference was given to contemporary studies reporting clinical or echocardiographic outcomes after leadless pacemaker implantation, while seminal earlier investigations were included to provide historical context.

### 2.1 TR After Leadless Pacemaker Implantation: Current Evidence

Multiple observational studies and meta‐analyses have evaluated TR severity following leadless pacemaker implantation. In the most comprehensive meta‐analysis, Haeberlin et al. pooled 297 patients across eight studies and found no significant change in TR severity following implantation (risk ratio 1.22, 95% CI 0.97–1.53) [5].

Nonetheless, clinically relevant worsening of TR still occurs. Sasaki et al. reported TR progression in 33% of patients with leadless pacemakers compared with 20% of those with transvenous systems (*p* = 0.04) [7]. In multivariable analysis, loss of AV synchrony was independently associated with TR progression and demonstrated the strongest association among the variables included in the model [7].

The observational design of Sasaki et al. does not permit differentiation between the acute hemodynamic consequences of AV dyssynchrony and progressive structural remodeling of the tricuspid annulus and right ventricle. It is likely that both mechanisms contribute, with acute alterations in ventricular filling and right‐sided pressures potentially initiating longer‐term remodeling. Serial imaging studies are required to clarify these temporal relationships.

Consistent with these findings, a large meta‐analysis by Yuyun et al. including over 13 000 patients demonstrated that leadless pacing was not associated with increased TR risk, whereas transvenous RV pacing significantly increased TR (OR 4.54) [8].

Arps et al. further showed that while TR severity remained stable, leadless pacing was associated with reductions in LVEF and TAPSE, reflecting the adverse hemodynamic effects of RV pacing [9]. Most available studies evaluating TR after leadless pacemaker implantation were conducted before the widespread adoption of Micra AV or combined Micra VR and Micra AV recipients without reporting device‐specific outcomes.

Consequently, although the existing literature consistently suggests that leadless pacing does not substantially increase TR overall, the independent effect of AV‐synchronous leadless pacing remains uncertain. This limitation underscores the need for prospective studies directly comparing Micra AV and Micra VR using standardized echocardiographic assessment of tricuspid valve function.

The relative contribution of these mechanisms likely varies according to patient characteristics, underlying RV function, pacing burden, and device programming, further emphasizing the multifactorial nature of pacing‐associated TR.

Implant location within the right ventricle may likewise influence ventricular activation patterns and potentially affect subsequent TR progression. Septal and apical implantation sites produce different electrical activation sequences, although current evidence remains limited and inconsistent, and the independent contribution of implant location to TR has not been clearly established [10].

### 2.2 Micra AV Versus Micra VR: Hemodynamic Differences

The primary hemodynamic distinction between Micra AV and Micra VR lies in the restoration (or absence) of AV synchrony. Micra VR provides single‐chamber ventricular pacing (VVI/VVIR mode), which frequently results in AV dyssynchrony when intrinsic atrial activity is present. In contrast, Micra AV utilizes an accelerometer‐based algorithm to detect atrial mechanical activity and enable VDD pacing, thereby approximating AV synchrony without a dedicated atrial lead.

The MARVEL 2 trial provided the most robust prospective evaluation of the Micra AV algorithm. In this multicenter study, 75 patients with previously implanted Micra devices underwent algorithm testing. Among 40 patients with sinus rhythm and complete AV block, the proportion achieving ≥ 70% AV synchrony at rest increased dramatically from 0% during VVI pacing to 95% during VDD pacing (*p* < 0.001) [6]. Mean AV synchrony improved from 26.8% to 89.2% with activation of the algorithm (Table 1).

Despite these promising findings, several important limitations constrain the interpretation of these results with respect to TR outcomes. First, the primary hemodynamic endpoint was left ventricular outflow tract velocity time integral (LVOT VTI), a left‐sided parameter that does not directly reflect right atrial pressure, tricuspid valve competence, or RV filling dynamics. Second, the study did not include echocardiographic assessment of TR severity before and after activation of AV‐synchronous pacing, precluding any direct inference regarding valvular outcomes. Third, AV synchrony was assessed under resting conditions, whereas real‐world ambulatory synchrony is substantially lower. Observational data suggest that ambulatory AV synchrony ranges from 29% to 75% following initial programming and may improve to 40%–86% after iterative optimization [11]. Furthermore, AV synchrony with Micra AV is dependent on consistent atrial mechanical sensing and may be reduced in patients with atrial arrhythmias, elevated atrial pressures, or suboptimal device positioning.

Additionally, AV synchrony declines at higher sinus rates (≥ 80 bpm), largely due to fusion of atrial and ventricular mechanical signals within the sensing window [11]. This limitation is particularly relevant in younger or more active patients. Finally, the definition of AV synchrony used in MARVEL 2—a P‐wave followed by a ventricular event within a 300 ms window—may overestimate true physiologic AV coupling, as it does not account for optimal timing of atrial contribution to ventricular filling.

Real‐world data from the Micra AV post‐approval registry further contextualize these findings. In this cohort, the median AV synchrony index was 79.4% (interquartile range 65.2%–86.4%) among patients with high ventricular pacing burden (> 90%) [12]. While these data support the feasibility of AV synchrony in clinical practice, they also highlight variability in performance and the potential gap between theoretical and effective synchrony.

Taken together, these data demonstrate that Micra AV consistently improves AV synchrony compared with Micra VR, but the extent to which this translates into meaningful differences in TR progression remains unknown.

It is important to distinguish AV synchrony from intraventricular synchrony. AV synchrony refers to coordinated timing between atrial contraction and ventricular activation, optimizing ventricular filling. In contrast, intraventricular synchrony describes coordinated activation within the ventricles through the His–Purkinje conduction system. Conventional RV pacing, including Micra VR and Micra AV, may preserve AV timing while still producing ventricular electrical and mechanical dyssynchrony. Conversely, CSP seeks to preserve physiologic ventricular activation and therefore addresses a distinct mechanism of pacing‐induced dysfunction.

**Table 1:** MARVEL 2 hemodynamic findings.

Parameter	VVI	VDD	Difference	*p* value
AV synchrony (%)	26.8	89.2	+62.4	< 0.001
Patients with ≥ 70% AV synchrony	0%	95%	—	< 0.001
LVOT VTI (% change)	—	+8.8%	—	< 0.05

*Note:* LVOT VTI is a left‐sided hemodynamic measure and does not directly assess TR severity or right‐sided pressures.

### 2.3 Loss of AV Synchrony as a Predictor of TR Worsening

Among currently available observational studies, Sasaki et al. provide some of the strongest evidence linking AV dyssynchrony with TR progression [7].

These findings suggest that preservation of AV synchrony may be an important determinant of TR progression independent of device platform, although this hypothesis requires prospective validation.

### 2.4 Comparison With CSP

Emerging evidence suggests that CSP, particularly pacing targeting the left bundle branch area, may represent a more physiologic alternative to both conventional RV pacing and leadless ventricular pacing by preserving native His–Purkinje activation and minimizing electrical and mechanical dyssynchrony. However, the available comparisons are observational, and randomized studies directly comparing CSP with leadless pacing for TR outcomes are not yet available.

Zhou et al. evaluated 386 patients across four pacing modalities—RV apical pacing, leadless pacing, deep septal pacing, and left bundle branch area pacing—and demonstrated that both leadless pacing and deep septal pacing were independently associated with higher odds of TR worsening compared with left bundle branch area pacing (OR 2.41 and 2.44, respectively; both *p* = 0.01) [13]. Moreover, left bundle branch area pacing was associated with more favorable preservation of left ventricular function, with a mean LVEF change of −1% compared with −5% in the leadless pacing group (*p* < 0.01).

Similarly, Vijayaraman et al. performed a propensity‐matched comparison of leadless pacing, conventional RV pacing, and CSP, demonstrating lower rates of death or heart failure hospitalization with CSP, particularly among patients with a high ventricular pacing burden (> 40%) [14]. These findings underscore the importance of physiologic ventricular activation in maintaining ventricular synchrony and minimizing adverse remodeling.

Comparative data between CSP and biventricular pacing (cardiac resynchronization therapy, CRT) with respect to TR are limited. CRT has been associated with improvement in functional TR, largely mediated by left ventricular reverse remodeling and reduction in tricuspid annular dilation [15, 16]. However, CRT does not restore physiologic ventricular activation and does not directly address RV dyssynchrony. In contrast, CSP preserves native conduction and promotes coordinated interventricular activation.

Although TR‐specific outcomes have not been systematically evaluated in direct comparisons, these mechanistic differences suggest that CSP may provide incremental benefit over both conventional RV pacing and CRT in mitigating TR progression. Prospective studies directly comparing CSP, CRT, and leadless pacing with standardized echocardiographic assessment of TR are warranted.

### 2.5 Comparison With Dual‐Chamber Leadless and Conventional Systems

A key limitation of currently available leadless pacemakers is that both Micra VR and Micra AV rely on a single ventricular device. While Micra AV provides AV synchrony through mechanical atrial sensing, it does not offer true atrial pacing capability and remains dependent on intrinsic atrial activity.

Recently, true dual‐chamber leadless pacing has been introduced with the Aveir DR leadless pacemaker system. This system consists of separate right atrial and RV leadless devices that communicate wirelessly, enabling direct atrial pacing and coordinated AV synchrony.

In the pivotal study published in the New England Journal of Medicine, dual‐chamber leadless pacing demonstrated high procedural success, reliable device‐to‐device communication, and effective AV synchrony in the majority of patients [17]. This represents the first leadless system capable of approximating the functionality of a conventional dual‐chamber transvenous pacemaker without the need for transvalvular leads or a subcutaneous generator pocket.

From a TR perspective, dual‐chamber leadless pacing is particularly relevant because it may address both principal mechanisms of device‐related TR. First, by eliminating transvalvular leads, it avoids direct mechanical leaflet interference, which is a well‐established contributor to TR in conventional systems. Second, by enabling true dual‐chamber pacing, it may mitigate AV dyssynchrony more effectively than Micra AV, which relies on indirect atrial sensing and may have variable synchrony in real‐world conditions.

In contrast, conventional dual‐chamber transvenous pacemakers provide highly reliable AV synchrony and extensive programming flexibility but are associated with lead‐related complications, including lead‐induced TR due to direct interaction with the tricuspid valve apparatus [19].

When considered alongside existing leadless systems, important distinctions emerge. Micra VR provides ventricular‐only pacing and is therefore associated with complete AV dyssynchrony in patients with preserved atrial activity. Micra AV provides partial restoration of AV synchrony through accelerometer‐based atrial sensing but remains dependent on intrinsic atrial activity and exhibits variable synchrony in ambulatory conditions. In contrast, the Aveir DR leadless pacemaker system enables true dual‐chamber pacing with direct atrial and ventricular coordination. From a mechanistic standpoint, this places Aveir DR closest to conventional dual‐chamber pacing in its ability to preserve physiologic AV coupling while avoiding transvalvular leads.

Taken together, these observations suggest that different pacing strategies preserve different components of cardiac synchrony. Conventional dual‐chamber transvenous pacing and dual‐chamber leadless pacing primarily preserve AV synchrony, whereas CSP additionally preserves physiologic ventricular activation through the native His–Purkinje system. Micra AV restores AV synchrony without correcting RV activation patterns, while Micra VR provides ventricular pacing without AV synchrony in patients with intact atrial activity. These approaches therefore address complementary rather than equivalent mechanisms of pacing‐induced dyssynchrony, and direct comparative studies evaluating their effects on TR are needed.

### 2.6 Real‐World Safety Data

Large real‐world registries consistently demonstrate a favorable safety profile for leadless pacemakers. The Micra AV Coverage with Evidence Development (CED) study, which included 7552 patients, showed that at 2 years, Micra AV implantation was associated with significantly lower rates of major complications compared with dual‐chamber transvenous pacemakers (adjusted 5.3% vs. 9.6%; HR 0.54, 95% CI 0.49–0.61) [11]. Similarly, re‐intervention rates were lower in the leadless cohort (adjusted 3.5% vs. 5.6%; HR 0.62, 95% CI 0.54–0.72) [11].

The Micra AV post‐approval registry further supported these findings, reporting a 3.7% major complication rate and a 1.5% system revision rate at 12 months, both significantly lower than historical transvenous controls [12].

Comparable findings have been observed with the Micra VR system. In a large cohort analysis, leadless ventricular pacing demonstrated similar or lower complication rates compared with transvenous single‐chamber systems, with particularly low infection rates (~0.2%) [18].

It is important to note that all‐cause mortality appears higher among patients receiving leadless pacemakers in some observational analyses (adjusted HR ~1.5), likely reflecting greater baseline comorbidity burden rather than device‐related risk [11] (Table 2).

**Table 2:** Safety outcomes of leadless pacemakers.

Study	*N*	Follow‐up	Major complications	System revision	Infection rate
Micra AV CED [11]	7552	2 years	5.3%	3.5%	0.2%
Micra AV PAR [12]	796	12 months	3.7%	1.5%	0.2%
Micra VR CED [18]	5746	22 months	5.0%	—	0.2%

*Note:* Values represent study‐level outcomes and are not derived from direct head‐to‐head comparisons.

Leadless pacemakers demonstrate consistently low complication and infection rates compared with transvenous systems.

### 2.7 Limitations of Current Evidence

Several limitations of the current evidence should be acknowledged:

1. No direct Micra AV versus Micra VR TR comparison,
2. Predominantly observational data,
3. Lack of standardized echocardiographic assessment,
4. Overestimation of AV synchrony in controlled settings,
5. TR still occurs despite leadless design [5, 7],
6. CSP may be superior [13, 14],
7. Implant location effects incompletely understood [10].

### 2.8 Future Directions

Future studies should include:

- Randomized comparisons (Micra AV vs. Micra VR),
- Standardized TR assessment with core labs,
- Long‐term follow‐up,
- Inclusion of dual‐chamber leadless systems,
- Clinical outcomes beyond echocardiography.

## 3 Conclusions

Leadless pacemakers eliminate transvalvular leads and therefore reduce mechanical contributors to TR, but do not eliminate functional mechanisms of TR progression. Current evidence suggests that AV dyssynchrony represents one important and potentially modifiable contributor to pacing‐associated functional TR; however, its effects should be considered within the broader context of intraventricular dyssynchrony, papillary muscle discoordination, RV pacing burden, RV dysfunction, and progressive right‐heart remodeling. The relative contribution of each mechanism remains incompletely defined.

Among currently available leadless systems, the degree of achievable AV synchrony varies substantially, with Micra AV providing AV synchrony through atrial mechanical sensing and Aveir DR enabling true dual‐chamber leadless pacing. Preservation of AV synchrony should be distinguished from preservation of physiologic ventricular activation, the latter being a principal advantage of CSP. These pacing strategies, therefore, address different aspects of cardiac synchrony rather than representing equivalent physiologic approaches.

Prospective studies incorporating standardized echocardiographic assessment, longer‐term follow‐up, and direct comparisons among Micra VR, Micra AV, dual‐chamber leadless systems, conventional dual‐chamber pacing, and CSP are needed to define the optimal pacing strategy to minimize TR and improve long‐term clinical outcomes.

## Author Contributions

**Zachary Alvis Seal:** conceptualization, literature search, writing – original draft, and writing – review and editing. **Debanshu Roy, Jorge Alejandro Irizarry Caro, Ivan Alfredo Mijares Rojas, Osamah Basil Ibrahim Altaee**, and **Binish Qureshi:** literature review and critical revision of the manuscript. **Robert Chilton:** supervision and critical revision of the manuscript. All authors reviewed and approved the final manuscript.

## Funding

The authors have nothing to report.

## Ethics Statement

This article is a narrative review of previously published literature and did not involve the collection of new data from human participants, animals, or identifiable private information. Therefore, ethics committee or institutional review board approval was not required. Patient consent was not applicable.

## Conflicts of Interest

The authors declare no conflicts of interest.

## Data Availability

Data sharing is not applicable to this article because no new data sets were generated or analyzed. This narrative review synthesizes data from previously published studies.
